# Clinical efficacy of laminectomy with instrumented fixation in treatment of adjacent segmental disease following ACCF surgery: a retrospective observational study of 48 patients

**DOI:** 10.1038/s41598-019-43114-9

**Published:** 2019-04-25

**Authors:** Sidong Yang, Dalong Yang, Lei Ma, Hui Wang, Wenyuan Ding

**Affiliations:** 1 Department of Spinal Surgery, The Third Hospital of Hebei Medical University, Shijiazhuang, 050051 China; 2 Hebei Provincial Key Laboratory of Orthopedic Biomechanics, Shijiazhuang, 050051 China

**Keywords:** Trauma, Bone, Risk factors, Outcomes research

## Abstract

This study was designed to investigate the clinical efficacy of laminectomy with instrumented fixation in treatment of adjacent segmental diseases following anterior cervical corpectomy and fusion (ACCF) surgery. Between January 2008 and December 2015, 48 patients who underwent laminectomy with instrumented fixation to treat adjacent segmental diseases following ACCF surgery, were enrolled into this study. The patients were followed up at least 2 years. Pain assessment was determined by visual analogue scale (VAS) score and Neck Disability Index (NDI) score; neurological impairment was evaluated by Japanese Orthopaedic Association (JOA) score; and radiographic parameters were also compared. All comparisons were determined by paired t test with appropriate Bonferronni correction. VAS score preoperatively and at last follow-up was 5.28 ± 2.35 *vs* 1.90 ± 1.06 (P < 0.001). JOA score preoperatively and at last follow-up was 8.2 ± 3.6 *vs* 14.5 ± 1.1 (P < 0.001). NDI score preoperatively and at last follow-up was 30.5 ± 12.2 *vs* 10.6 ± 5.8 (P < 0.001). Moreover, the losses of cervical lordosis and C2-C7 range of motion after laminectomy were significant (both P < 0.005), but not sagittal vertical axis distance. Postoperative complications were few or mild. In conclusion, clinical effectiveness and safety can be guaranteed when the patients undergo laminectomy with instrumented fixation to treat adjacent segmental diseases following ACCF surgery.

## Introduction

Anterior cervical discectomy and fusion (ACDF) and anterior cervical corpectomy and fusion (ACCF) have been considered as the “gold standard” surgical treatment of cervical degenerative diseases. However, data from radiographic and clinical studies showed that the segments adjacent to the fused spinal segments would accelerate the progression of degeneration or become unstable after a certain years^1–3^.

Both ACDF and ACCF surgery have changed the original mechanical behavior of the cervical spine at the cost of the activity of the fused level, which is likely to cause the changes of adjacent vertebral stress distribution and the movement patterns, resulting in biomechanical changes including stress concentration of adjacent segments, compensatory increase in activity, and even instability; finally, adjacent segmental disease (ASD) developed^4–6^. Once the ASD progressed to a severe extent, the patients would usually undergo another surgery performed via a posterior way, including laminoplasty and laminectomy. However, both laminoplasty and laminectomy without fixation are likely to aggravate sagittal imbalance and contribute to the progression of cervical kyphosis^7–10^. Therefore, laminectomy with instrumented fixation would be better than that without any fixation when a second surgery is performed via a posterior way. To date, there have been few studies reporting clinical effectiveness in regards to posterior laminectomy with instrumented fixation in treating ASD following ACDF or ACCF.

In this study, to minimize the confounding factors, it was designed to investigate the clinical effectiveness of laminectomy with instrumented fixation in treating ASD following only ACCF, based on a regular follow-up with a minimum of two years.

## Patients and Methods

### Ethical statement

This study was approved by Ethics Committee of The Third Hospital of Hebei Medical University (Approval Number: KY2018-05-001). Informed consent was obtained from all participants and/or their legal guardian/s. The methods were carried out in accordance with the relevant guidelines and regulations.

### Patient selection

**Figure 1:**
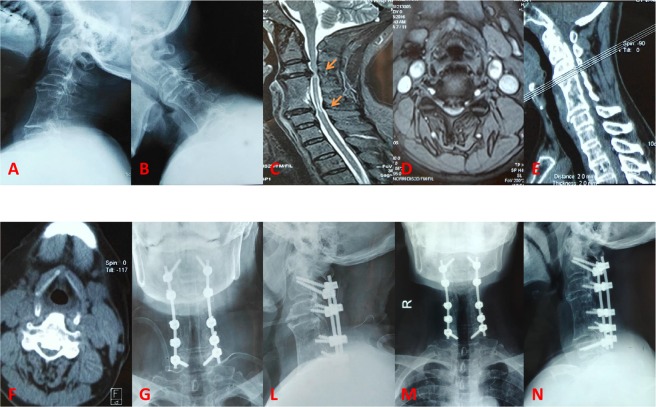
Radiological images preoperatively and postoperatively. (**A**,**B**) preoperative X-ray images; (**C**,**D**) preoperative MRI scan; (**E**,**F**) preoperative CT scan; (**G**,**L**) postoperative X-ray immediately; (**M**,**N**) postoperative X-ray at final follow-up.

In this study, all identified patients have undergone posterior cervical laminectomy and instrumented fixation with a history of previous ACCF surgery, as shown in Fig. 1. All patients were regularly followed up after surgery, at the timepoint of 1 week, 3 months, 1 year and thereafter.

### Surgical procedures

Posterior cervical laminectomy and instrumented fixation was performed as follows. Briefly, a standard posterior approach was performed. In the first place, an incision was performed in the midline of the neck. Second, we separated the muscle along C2-C7 to expose spinous process, articular process, vertebral arch and laminae. In addition, two pedicle screws were implanted into C2 and C7, respectively. And then, two lateral mass screws were implanted into C3, C4, C5, and C6, respectively (where adjustment could apply). At last, we performed the laminectomy of C2-C7 using a ball mill drill to achieve thorough decompression of cervical spinal canal. Then two connecting rods were used to connect the screws together in both sides.

### Radiographic measurements

Radiographic parameters were collected by screening neutral and dynamic flexion-extension lateral radiographs, including C2-C7 sagittal vertical axis (SVA) distance, cervical lordosis and range of motion (ROM). Neutral-position and dynamic flexion-extension lateral radiographs during each follow-up examination were evaluated with the PACS software and a PACS workstation (Centricity 2.0, General Electrics Medical Systems, Milwaukee, WI).

### Assessment of clinical effectiveness

All patients were required to return our hospital for regular follow-up after surgery. Clinical and radiological evaluations were performed postoperatively, at 1 week, 3 months, 1 year, and last follow-up (more than 2 years). Clinical effectiveness was assessed by visual analogue scale (VAS) score, Japanese Orthopaedic Association (JOA) score (17 points system, 1994 revised edition) and Neck Disability Index (NDI) score. Specifically, pain assessment was determined by visual analogue scale (VAS) score and Neck Disability Index (NDI) score; neurological impairment was evaluated by Japanese Orthopaedic Association (JOA) score. The recovery rate (RR) of JOA score was calculated according to the following formula: RR = (postoperative scores − preoperative scores)/(17 − preoperative scores) * 100%.

### Statistical analysis

SPSS for windows (version 18.0, IBM SPSS Inc., Chicago, USA) was applied to perform statistical analyses. Data are presented as Mean ± SD (standard deviation) for measurement data. Comparisons of VAS, JOA, NDI score, and radiographic parameters between pre- and post-surgery were determined by paired t test. P < 0.05 with appropriate Bonferronni correction was defined as significant.

## Results

### Baseline and surgical data

Between January 2008 and December 2015, 48 patients who underwent laminectomy with instrumented fixation to treat adjacent segmental diseases following ACCF surgery, were identified and enrolled into this study. In total, there were 26 males and 22 females. The patient age was 61 ± 17 years. The duration between previous ACCF and posterior laminectomy was 16 ± 11 years. The surgery took 160 min on average. Blood loss was 700 ml on average. Median blood transfusion was 400 ml (ranging from 200 to 800 ml). All cases completed regular follow-up of a minimum of 2 years, with an average of 38 months. Postoperative complications were few or mild. Five patients sustained lower-limb vein thrombosis but asymptomatic. Two patients suffered C5 palsy which was finally diminished. Besides, two patients experienced delayed wound healing but finally well healed.

### VAS score

As shown in Table 1, VAS score preoperatively was 5.28 ± 2.35, and 1.90 ± 1.06 postoperatively at last follow-up. Statistically, VAS score achieved significant improvement compared with the preoperative scores (P < 0.001).

**Table 1:** Comparison regarding VAS score.

preoperatively	1 week postoperatively	3 months postoperatively	1 year postoperatively	Last follow-up	Statistics
5.28 ± 2.35	3.91 ± 2.31	2.85 ± 1.29	1.94 ± 1.12	1.90 ± 1.06	
	2.88	6.28	8.89	9.08	t
	0.005	<0.001	<0.001	<0.001	p*

*Compared with VAS score preoperatively. VAS, visual analogue scale.

### JOA score and RR

As shown in Table 2, JOA score preoperatively was 8.2 ± 3.6, and 14.5 ± 1.1 postoperatively at last follow-up. Statistically, the difference was significant regarding JOA score compared with the preoperative scores (P < 0.001). In addition, RR was calculated to be 70.58% ± 4.6% at last follow up.

**Table 2:** Comparison regarding JOA score.

preoperatively	1 week postoperatively	3 months postoperatively	1 year postoperatively	Last follow-up	Statistics
8.2 ± 3.6	10.1 ± 2.2	13.2 ± 1.6	14.3 ± 1.0	14.5 ± 1.1	
	3.12	8.79	11.31	11.60	t
	0.0024	<0.001	<0.001	<0.001	p*

*Compared with JOA score preoperatively. JOA, Japanese Orthopaedic Association.

### NDI score

As shown in Table 3, NDI score preoperatively was 30.5 ± 12.2, and 10.6 ± 5.8 postoperatively at last follow-up. Statistically, the difference was significant regarding NDI score compared with the preoperative scores (P < 0.001).

**Table 3:** Comparison regarding NDI score.

preoperatively	1 week postoperatively	3 months postoperatively	1 year postoperatively	Last follow-up	Statistics
30.5 ± 12.2	24.5 ± 4.5	16.6 ± 5.4	11.0 ± 6.5	10.6 ± 5.8	
	3.20	7.22	9.77	10.21	t
	0.0019	<0.001	<0.001	<0.001	p*

*Compared with NDI score preoperatively. NDI, Neck Disability Index

### Radiographic parameters

As shown in Table 4, preoperative cervical lordosis was 12.5 ± 6.3 while it was 8.7 ± 6.0 postoperatively at last follow-up (P = 0.003). As shown in Table 5, C2-C7 ROM decreased after laminectomy to 25.6 ± 9.5, from 43.6 ± 5.9 preoperatively (P < 0.001). As shown in Table 6, preoperative SVA distance was 28.05 ± 5.4 mm, and it increased to 29.15 ± 5.6 mm postoperatively at last follow-up, but without any statistical significance (P = 0.330).

**Table 4:** Comparison regarding cervical lordosis.

preoperatively	1 week postoperatively	3 months postoperatively	1 year postoperatively	Last follow-up	Statistics
12.5 ± 6.3	12.1 ± 6.8	11.8 ± 6.2	10.2 ± 5.8	8.7 ± 6.0	
	0.30	0.55	1.86	3.03	t
	0.766	0.585	0.066	0.003	p*

*Compared with cervical lordosis preoperatively.

**Table 5:** Comparison regarding C2-C7 ROM.

preoperatively	1 week postoperatively	3 months postoperatively	1 year postoperatively	Last follow-up	Statistics
43.6 ± 5.9	32.5 ± 5.9	33.8 ± 6.3	28.6 ± 7.5	25.6 ± 9.5	
	9.22	7.87	10.89	11.15	t
	<0.001	<0.001	<0.001	<0.001	p*

*Compared with C2-C7 ROM preoperatively. ROM, range of motion.

**Table 6:** Comparison regarding C2-C7 SVA.

preoperatively (mm)	1 week postoperatively	3 months postoperatively	1 year postoperatively	Last follow-up	Statistics
28.05 ± 5.4	28.09 ± 6.5	28.20 ± 5.9	28.56 ± 4.4	29.15 ± 5.6	
	0.03	0.13	0.51	0.98	t
	0.974	0.897	0.613	0.330	p*

*Compared with C2-C7 SVA preoperatively. SVA, sagittal vertical axis.

## Discussion

In a clinical setting, ACDF and ACCF are widely used surgical procedures by neurosurgeons and spine surgeons in treatment of cervical spondylosis. However, data from radiographic and clinical studies showed that the segments adjacent to the fused spinal segments would accelerate the progression of degeneration or become unstable after a certain years. Both ACDF and ACCF would yield the loss of moter function in the fused level, which is likely to cause the changes of adjacent vertebral stress distribution and the movement patterns, resulting in biomechanical changes including stress concentration of adjacent segments, compensatory increase in activity, and even instability; finally, ASD may develop to a severe degree. As such, the patients are more likely to undergo a posterior surgery, such as laminoplasty and laminectomy.

As reported previously, laminoplasty, laminectomy, and laminectomy with instrumented fixation or fusion, all have improved cervical function in the near term^11,12^. The shortcoming of posterior approach (without thorough decompression and fusion) is that ventral compression may persist if the backward drift of the spinal cord is not enough, resulting in an unsatisfactory neurofunctional recovery. Laminectomy has shown a close association with late deterioration of kyphosis, segmental instability and neurological deterioration, compared with the other posterior surgery^12^. Laminoplasty should be avoided as a preferred approach in treating patients with preoperative kyphosis or instability, but it seems safer and less trauma and economic burden. Thus, for patients with adequate stabilization and lordosis, laminoplasty could be a good alternative, because laminoplasty can preserve the motor function of motor segments by widely decompressing, which is in line with the current concept of non-fusion. However, both laminoplasty and laminectomy (if without fixation) are likely to aggravate sagittal imbalance and contribute to the progression of cervical kyphosis^7–10,12^. Hence, laminectomy with instrumented fixation appears to be a better surgical procedure when a posterior operation is scheduled for ASD (especially when more than two levels) following a previous anterior operation, such like ACDF and ACCF.

Over the past few years, laminectomy has been shown as an effective and safe technique in treatment of multilevel cervical spondylotic myelopathy^13–15^. However, accumulating evidence has revealed increased rate of approach-related complications caused by the posterior approach as compared with the anterior approach, particularly in multilevel surgery^16–19^. It is well known that postoperative C5 palsy is not rare after cervical surgery. Although there remains controversy, C5 palsy is considered to be more common in patients who had laminectomy and fusion than those who had laminoplasty. However, the reason for the higher incidence of C5 palsy in patients with laminectomy and fusion has been poorly understood. A recent study^20^ reported a high occurrence rate of 14.3% with C5 palsy and verified that C4-C5 foraminal stenosis was the only risk factor for C5 palsy after laminectomy. In our study, only two patients suffered C5 palsy after laminectomy, which was a much lower incidence rate.

By contrast to the posterior approach, the advantage of anterior approach is apparent; it is the most effective technique in a situation where a direct surgical decompression is urgently needed because it can directly remove such compressive anterior components as osteophytes, disc herniations or ossification of posterior longitudinal ligament^21,22^. Previously, it has been indicated that single posterior laminectomy would be insufficient in terms of decreasing intramedullary pressure^23^. In contrast, neurological deterioration due to increased intramedullary pressure may result from progressive kyphosis which can be caused by non-instrumented laminectomy^9^. Nevertheless, laminectomy remains to be a valuable surgical technique for decompression of cervical spinal canal, particularly when combined with instrumented fixation and fusion using lateral mass screws in order to prevent postoperative instability and progression of kyphosis. Therefore, cervical laminectomy with instrumented fixation/fusion is believed to be the best choice for ASD following ACDF/ACCF. In the current study, we only focus on the treatment of ASD due to ACCF, excluding the type caused by ACDF, because ACDF and ACCF are different in terms of fusion levels, which is possibly a confounding factor to the further analysis. Thus, to minimize such a confounding factor, we here only investigated the clinical efficacy of laminectomy with instrumented fixation in treatment of ASD following ACCF.

To the best of our knowledge, there have been no reports on assessment of clinical outcomes of laminectomy with instrumented fixation in treatment of ASD following ACCF. Adjacent segment degeneration and related diseases have become major concerns following anterior fusion surgery. The data from a recent meta-analysis has shown that the pooled incidence of adjacent segment degeneration following a cervical fusion surgery is 32.8%, and approximately 1/4-1/3 of such degeneration would progress to ASD in the future^24^. Apparently, the incidence of ASD is really high; almost one of ten cases undergoing ACDF/ACCF surgery would progress to ASD some day. Thus, it is important to clarify the clinical efficacy and safety of laminectomy with instrumented fusion in treatment of ASD.

In this study, all of the neurological function improvements appear satisfying, with regard to JOA score, VAS score, and NDI score. It is consistent with the reported studies that have confirmed such improvements due to laminectomy with or without fusion^25,26^. As reported by recent meta-analysis^25,26^, laminectomy with fusion and laminoplasty yield similar results regarding the loss of cervical lordosis. In our study, only cervical lordosis at last follow-up is statistically lower than the preoperative one. Thus, the loss of lordosis due to laminectomy with instrumented fixation is progressive, but slower than the others. During the surgical procedures for the patients, the facet joints have been damaged by ball mill drill that we used to assist resection of the vertebral laminae. Owing to that, posterior fusion of cervical spine has formed, making the whole cervical structure more stable.

This study goes along with some limitations. First of all, the retrospective character of the study design was bound to cause some selection bias. In addition, this study was only an observational study; a comparative study would be better. Lastly, the small sample size was also a limitation in this study. Therefore, a randomized clinical trial with a large sample is needed to further clarify the clinical outcomes in the future.

## Conclusions

In summary, clinical effectiveness and safety can be guaranteed when the patients undergo laminectomy with instrumented fixation to treat adjacent segmental diseases following ACCF surgery.

## Data Availability

Data in this study are available.
